# P-1262. Safety and Pharmacokinetics of Ainuovirine, a Novel Non-NucleosideReverse Transcriptase Inhibitor, in Healthy Chinese Adults

**DOI:** 10.1093/ofid/ofae631.1444

**Published:** 2025-01-29

**Authors:** Hao Wu, Meixia Wang, Bin Su, Juan Yang, Xinming Yun, Hong Qin

**Affiliations:** Beijing Youan Hospital Affiliated to Capital Medical University, Beijing, Beijing, China; Beijing Youan Hospital Affiliated to Capital Medical University, Beijing, Beijing, China; Beijing Youan Hospital Affiliated to Capital Medical University, Beijing, Beijing, China; Jiangsu Aidea Pharmaceutical Co., Ltd, Nanjing, Jiangsu, China; Jiangsu Aidea Pharmaceutical Co., Ltd, Nanjing, Jiangsu, China; Jiangsu Aidea Pharmaceutical Co., Ltd, Yangzhou, Jiangsu, China (People's Republic)

## Abstract

**Background:**

Ainuovirine is a novel non-nucleoside reverse transcriptase inhibitor (NNRTI) for treatment of human immunodeficiency virus type 1 (HIV-1) infection. The safety profile and pharmacokinetics of ainuovirine was characterized in healthy adults administered with single ascending dose.

**Table 1.**
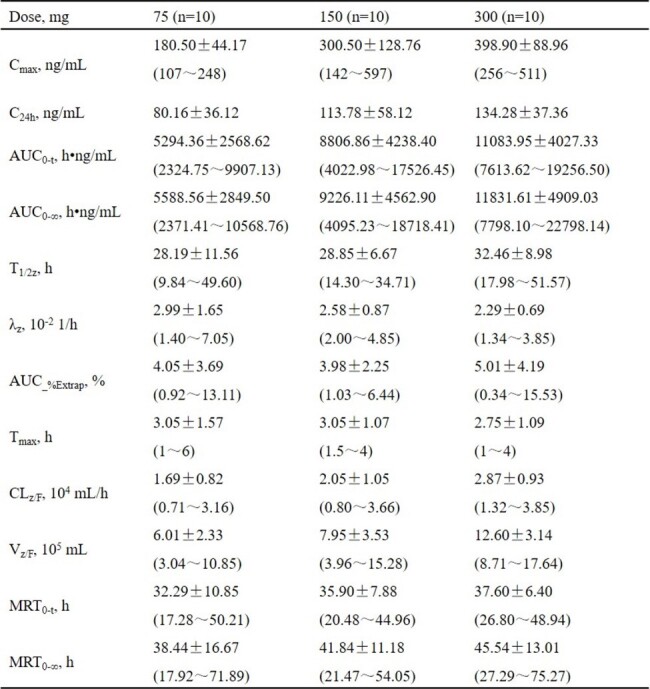
Summary statistics of pharmacokinetic parameters of ainuovirine after single oral doses in healthy participants.

Data are expressed in median ± standard deviation (min, max); AUC0–t, area under the plasma concentration-time curve from time zero to time of the last quantifiable concentration; AUC0–∞, AUC from time zero to infinity; AUC_%Extrap, percentage of extrapolated AUC; Cmax, maximum plasma concentration; C24h, concentrations at 24 hours postdose; CLz/F, apparent clearance; MRT0-t, mean retention time from time zero to time of the last quantifiable concentration; MRT0-∞, MRT from time zero to infinity; Vz/F, apparent volume of distribution; T1/2z, plasma terminal half-life; Tmax, time to maximum plasma concentration; λz, elimination rate constant.

**Methods:**

A single-center, open-label, single-dose escalation study was conducted among healthy Chinese adults. Thirty participants, aged from 18 to 45 years, were randomly allocated to each cohort given a single-dose ainuovirine 75, 150 or 300 mg (10 participants per cohort), respectively. Inhibitory quotient (IQ) was defined as the ratio of the clinical trough concentration (C_24h_) to the protein-binding adjusted 50% effective concentration (pa-EC_50_).

**Results:**

Across all dosage groups, most adverse events were rated as mild in severity. Major reported adverse events were abnormal serum lipids and elevated liver transaminase. No rash was reported and only one participant experienced central nervous system events (150 mg dose group). Pharmacokinetic parameters are shown in Table 1 for single-dose ainuovirine. Ainuovirine was readily absorbed, with a median T_max_ of approximately 3 hours. Ainuovirine exposure (C_max_ and AUC) increased to a lesser extent than the dose proportionality. Median apparent terminal half-life (T_1/2z_) remained similar across three dose cohorts, slightly longer than 24 hours. The IQs for wild type (pa-EC_50_=4.78 ng/mL) were 16.8, 23.8, and 28.1 folds, respectively. Those were 2.0, 2.9, and 3.4 folds for K103N (pa-EC_50_=39.17 ng/mL), respectively, and 1.1, 1.5, and 1.8 folds for Y181C (pa-EC_50_=232.2 ng/mL), respectively. All the IQs were above 1 fold.

**Conclusion:**

Ainuovirine was well tolerated and showed a dose-dependent,nonlinear pharmacokinetics. The pharmacikinetics supported once daily oral dosing regimen of ainuovirine, with candidate doses of 75 to 300 mg in subsequent dose-ranging, proof-of-concept study.

**Disclosures:**

**Juan Yang, M.S.**, Jiangsu Aidea Pharmaceutical Co., Ltd.: Honoraria **Xinming Yun, PhD**, Jiangsu Aidea Pharmaceutical Co., Ltd.: Honoraria **Hong Qin, MD, PhD**, Jiangsu Aidea Pharmaceutical Co., Ltd: Honoraria

